# Predictors of voluntary and compulsory admissions after psychiatric emergency consultation in youth

**DOI:** 10.1007/s00787-020-01558-9

**Published:** 2020-05-21

**Authors:** Pety So, André I. Wierdsma, Marianne C. Kasius, Jurgen Cornelis, Marion Lommerse, Robert R. J. M. Vermeiren, Cornelis L. Mulder

**Affiliations:** 1 Youz, Center for Youth Mental Healthcare, Rotterdam, The Netherlands; 2 Parnassia Psychiatric Institute, Rotterdam, The Netherlands; 3 Erasmus MC, University Medical Center, Epidemiological and Social Psychiatric Research Institute and Department of Psychiatry, Rotterdam, The Netherlands; 4 Youz, Center for Youth Mental Healthcare, The Hague, The Netherlands; 5 Department of Research, Arkin Mental Health Care, Amsterdam, The Netherlands; 6 Department of Emergency Psychiatry, Arkin Mental Health Care, Amsterdam, The Netherlands; 7 Department of Child and Adolescent Psychiatry, Curium-LUMC, Leiden University Medical Center, Leiden, The Netherlands

**Keywords:** Child psychiatry, Emergency admission, Predictors of hospitalization, Emergency mental health services, Intensive home treatment

## Abstract

As hospital beds are scarce, and emergency admissions to a psychiatric ward are major life-events for children and adolescents, it is essential to have insight into the decision-making process that leads to them. To identify potentially modifiable factors, we, therefore, studied the contextual and clinical characteristics associated with the voluntary and compulsory emergency admission of minors. We used registry data (2008–2017) on 1194 outpatient emergencies involving children aged 6–18 who had been referred to the mobile psychiatric emergency service in two city areas in The Netherlands. Demographic and contextual factors were collected, as well as clinical characteristics including diagnoses, psychiatric history, Global Assessment of Functioning (GAF), and the Severity of Psychiatric Illness (SPI) scale. Logistic regression analyses were used to identify factors that predict voluntary or compulsory admission. Of 1194 consultations, 227 (19.0%) resulted in an admission, with 137 patients (11.5%) being admitted voluntarily and 90 (7.5%) compulsorily. Independently of legal status, the following characteristics were associated with admission: severity of psychiatric symptoms, consultation outside the patient’s home, and high levels of family disruption. Relative to voluntary admission, compulsory admission was associated with more severe psychiatric problems, higher suicide risk, and prior emergency compulsory admission. Two potentially modifiable factors were associated with psychiatric emergency admission: the place where patients were seen for consultation, and the presence of family problems. Psychiatric emergency admissions may be reduced if, whenever possible, minors are seen in their homes and if a system-oriented approach is used.

## Introduction

Even though hospitalization it is a major life event, it is sometimes necessary, especially to stabilize and treat young people in a psychiatric crisis. While the overall prevalence of mental health conditions in youth has remained relatively stable [1], many recent studies have reported an increase in psychiatric emergencies in children and adolescents. Such increase has been noted in the United States [2–5], Canada [6–8], and Europe [9–11]. Over the same period, most countries have reduced the number of child and adolescent psychiatric inpatient beds [12–14]. Together with this reduction in inpatient beds, the increase in psychiatric emergencies makes it important to identify factors leading children and adolescents in an emergency situation to be admitted to a psychiatric hospital. Possible modifiable factors associated with emergency admission in youth should be identified, as these factors may be a target for preventing these admissions.

Various studies have identified demographic characteristics and clinical factors as predictors of voluntary admission of young patients seen in psychiatric crisis situations [15–18]. Demographic characteristics that were found to be associated with decisions to hospitalize children and adolescents after emergency consultation were relatively older age, minority status, and having been adopted [16–19]. Clinical factors that were associated with a higher chance of admission were suicide attempt or self-injurious behavior, the clinician’s appraisal of high suicide risk, the presence of a depressive or bipolar disorder, the severity of psychiatric symptoms, comorbidity, a lower score on the global assessment of functioning (GAF), and prior admission. The likelihood of hospitalization was also increased by a prior emergency consultation, substance abuse or dependence, and being on psychotropic medication [15–19]. However, both these demographic characteristics and the clinical factors cannot be modified to prevent emergency admissions.

While factors leading to voluntary admission have been described widely, we found no studies specifically describing factors leading to compulsory admission of minors in an emergency psychiatric situation. Studies comparing adolescents who had been admitted voluntarily to a psychiatric clinic with those who had been admitted compulsorily identified several clinical factors [19–21]. Compulsory detainment in adolescents was associated with symptom severity, mental retardation, substance abuse, and psychotic disorders. Compared to adolescents who had been admitted voluntarily, those who had been admitted compulsorily were also older, more often had black ethnicity, and more often had been admitted outside working hours [19–21].

To prevent emergency admissions in psychiatric crisis situations in youth with severe psychiatric problems, contextual characteristic may be an important target for home interventions. Some clinicians argue that psychiatric crisis situations are best dealt with in a patient’s home, where systemic interventions can take place directly within a child’s environment [22]. Family factors may influence decisions to admit a child to a psychiatric hospital after emergency consultation. Contextual characteristics that were found to be associated with the decision to hospitalize children and adolescents after emergency consultation were the absence of parents at consultation; the time and day of consultation (there being a greater chance of admission at night and in school weeks rather than during school holidays); and a rural location [15, 17, 18]. The source of referral was also associated with the decision to hospitalize: in cases of self-referral and referral by a family member or someone from a school, there was less chance of admission [16].

A systematic review of randomized-controlled trials reporting the efficacy of a variety of home-based interventions as alternatives to inpatient care in youth found these interventions to be associated with similar clinical improvement, greater patient satisfaction, and lower costs compared to inpatient care [23]. However, most studies in this review excluded children and adolescents with severe danger to self or others, so it is not clear if these results apply to children as well as adolescents with immediate and severe risks.

One study compared the effect of a special designed discharge service by an intensive home-treatment team to care as usual for youth admitted to a psychiatric hospital [24]. This study found no differences in inpatient days or treatment effect. However, patients in the intensive home-treatment group reported better school integration, more reduced self-harm, and less hospital use after discharge.

In literature, the proportion of psychiatric emergency consultations of minors that led to an admission ranges from 1.9 to 54.0% [15, 25]. As emergency psychiatric services for children and adolescents vary between countries [2, 6, 9, 15, 25], the comparison of the results is complicated. In The Netherlands, patients of all ages are examined at the patient’s location by outpatient psychiatric emergency services, which may offer an opportunity to intervene in some contextual characteristics.

Because most studies are based on data from children and adolescents in crisis that present to emergency departments (EDs), we aimed to identify factors upon which the outreaching emergency services in The Netherlands base their decisions on the emergency admission of minors. On the basis of previous research, we hypothesized that an important role in decisions to hospitalize children or adolescents after psychiatric emergency consultation may be played by contextual factors such as family support, living situation, and referral by the psychiatric services. We intended to identify factors that lead to the compulsory admission of young people in an emergency situation, as well. Due to the conditions imposed by Dutch law on compulsory admissions, we expected compulsory admission to be associated with the severity of psychiatric symptoms, danger to self or others, and lack of motivation for treatment.

## Method

### Setting

The study was conducted in two city areas in The Netherlands: Amsterdam (approximate population 800,000) and the Greater Rotterdam region (approximate population 1.2 million).

Outpatient psychiatric emergency services in The Netherlands are responsible 24 h a day for assessing any patients referred to them. Their primary tasks are triage and the subsequent referral of psychiatric emergency patients to other psychiatric services.

The childcare/custody protection service and regular mental healthcare providers in the Amsterdam region all receive emergency services for children and adolescents from the outreaching Mobile Crisis Team, which consists of junior psychologists specialized in the emergency care of young people and operates under the supervision of senior psychologists.

If a psychiatric emergency admission is considered, then, the patient is examined by a two-person team comprising a psychologist and a physician; a psychiatrist is consulted by telephone.

The staff of the outreaching emergency services in the greater Rotterdam region comprises community psychiatric nurses, physicians, and psychiatrists. Patients of all ages are examined at the patient’s location by a team consisting of a nurse and a physician or psychiatrist. If the physician is not a psychiatrist, a psychiatrist is consulted by telephone.

The training of the staff of the outreaching emergency services includes the assessment and treatment of addressing mental health problems in childhood and specifically on the management of crisis situations in emergencies.

In The Netherlands, self-referral and parent or school referral are not possible. In both regions, most patients are referred by general practitioners, police, the EDs at general hospitals, and mental healthcare workers. As police in The Netherlands are not permitted to take psychiatrically disturbed children to a psychiatric hospital, they usually ask for the emergency service staff to assess them at the police station.

The psychiatric emergency service assesses the patient, and, when applicable, their significant others. First, they try to resolve the crisis situation, preferably without hospitalization. However, if they decide that the crisis—such as severe suicidal behavior—is due to a psychiatric illness and that the patient cannot remain where he or she is—such as their parents’ home—he or she will be admitted to a psychiatric hospital. In The Netherlands, patients aged 12 years and older can be admitted against their will only if their mental disorder represents a danger to themselves or others, and when there are no alternatives to admission. The need for treatment alone is not a sufficient reason for compulsory admission [26].

A minor who is under the age of 12 can be admitted with the permission of his or her parents or legal guardian. Even if this is against his or her will, such a child may be admitted “voluntarily”. Under the Medical Treatment Agreements Act (WGBO), permission for this may be given by both parents who hold joint custody, one parent who holds sole custody, or by a legal guardian.

For adolescents aged between 12 but under 16, voluntary admission is subject to dual consent: his or her own, and that of the parents or legal guardian. If the patient does not agree, an application for emergency compulsory admission must be made to the city’s mayor.

For adolescents aged 16 years and older, solely, the adolescent has to consent to treatment; adult consent is not needed. If he or she does not want to be admitted, the emergency compulsory admission procedure must be followed. From the age of 18 onward, the rules for adults apply: if a psychiatrist decides that emergency compulsory admission is appropriate, a request must be submitted to the mayor, who can then order it.

### Patients

Data for the period from January 1, 2008 and January 1, 2017 were extracted from the records of the mobile psychiatric emergency services in the two city areas in question. We included all outpatient emergencies involving children aged 6–18 years whom a physician had referred for urgent consultation. The Medical Ethics Committee confirmed that the Medical Research Involving Human Subjects Act (Dutch acronym: WMO) did not apply to this study, and thus, that no informed consent was required.

### Data extraction

The study focused on the following variables:

*Demographic characteristics* These were collected for use in the analyses as non-modifiable control variables, and included age, gender, and living situation (two-parent family, single parent family, “other”, and unknown).

*Contextual characteristics* Place of consultation, referral source, time, and reasons for referral. Four place of consultation were defined: at home, at a police station, at the psychiatric services, and “other”. Examples of “other” locations are; at a general hospital, at a general practice center, in public space, and at other people’s homes. There were three referral sources: general practitioner, psychiatric services, and “other”. Time of referral was defined as daytime (8 am–6 pm); evening (6 pm–midnight); or night (midnight–8 am). Reasons for referral were defined as danger to self, danger to others, psychotic symptoms, anxiety/depressive symptoms, and “other”.

*Clinical characteristics* DSM-IV classifications, prior emergency consultation, treatment history, and severity of problems.

DSM-IV classifications were made by clinicians on the basis of a clinical interview and were registered in broad categories such as psychotic disorder and depressive disorders.

The primary DSM-IV Axis I classifications were grouped into four categories: internalizing disorders, externalizing disorders, relational problems and adjustment disorders; and “other”.

Axis II classifications: personality disorder (yes or no) and mental retardation (yes or no).

Both personality disorders and mental retardation were diagnosed only when sufficient information was available or when patients already had a diagnosis of personality disorder or mental retardation.

Prior emergency consultation was defined as a repeated emergency assessment within 12 months.

Treatment history was defined as currently being in outpatient psychiatric care (yes or no), having a history of psychiatric admission (yes or no), a history of emergency compulsory admission (yes or no), or a history of compulsory admission after a planned court order (yes or no). Compulsory admission using a court order is a planned compulsory admission that has been justified by a judge and arranged outside the context of an emergency psychiatric crisis situation.

Severity of problems was assessed using the GAF scale and the Severity of Psychiatric Illness Scale (SPI [27]; Dutch version by Mulder et al. [28]).

The SPI is a decision-support tool for assessing the need for services, especially those involving inpatient care. It provides a structured description of the severity of psychopathology and of possible complications regarding the disorder and regarding treatment. It has 14 items: suicide risk, danger to others, severity of symptoms, self-care ability, substance abuse or dependence, medical complications, family disruption, vocational impairment, residential instability, lack of motivation for treatment, lack of medical compliance, awareness of illness, lack of family involvement, and persistence of complaints.

These items were scored on a four-point scale from 0 (no problem) to 3 (severe problem). SPI ratings were dichotomized for the analyses: no/small problem and moderate/severe problem. The SPI is validated only for the use in adult patients, but is scored for every patient seen for emergency consultation. Because “self-care ability” and “awareness of illness” are dependent on developmental level and age, regardless of psychiatric illness or problems, these SPI items were excluded from the analyses. GAF scores were grouped as follows: 0–10, 10–20, 20–30, 30–40, etc.

### Analyses

Descriptive statistics were used to summarize the demographic, contextual, and clinical characteristics of all patients, those who had been admitted, those who had not been admitted, those who had been admitted voluntarily, and those who had been admitted compulsorily.

Correlation analyses were used to test the bivariate and partial associations between location of consultation, and GAF-score, SPI severity of symptoms, and SPI family disruption.

Multiple logistic regression analysis was used to identify variables related to admission and emergency compulsory admission. Following Hosmer & Lemeshow [29], we based variable selection on a stepwise procedure with *p* < 0.25 as entry level and *p* > 0.05 as removal level.

Model fit was assessed using Area-Under-the-Curve statistics and the Hosmer–Lemeshow goodness-of-fit test. In the final model, odds ratio estimates and their corresponding 95% confidence intervals were calculated for explanatory variables. All statistical analyses were performed using SPSS version 24.

## Results

Two hundred and twenty-seven of the 1194 consultations (19.0%) had resulted in an admission, 137 patients (11.5%) being admitted voluntarily and 90 (7.5%) being admitted compulsorily.

Table 1 presents the demographic and contextual characteristics. Most patients were girls; the mean age was 14.90 years (SD 2.3); the most common reason for referral was risk of suicide or self-harm, and most consultations had taken place in the daytime. While most children had been living with their parent(s), the living situation had been registered as unknown in 19.9% of the consultations. The second and third columns of Table 1 contrast the characteristics of the young people who had been admitted (whether voluntarily or compulsorily) against those of the young people who had not been admitted. Compared to the group of children who had not been admitted, the mean age of those admitted was higher, fewer were living with both parents, fewer consultations had taken place at home, and more consultations had taken place at night. The fourth and fifth columns of Table 1 contrast the characteristics of the young people who had been admitted voluntarily against those who had been admitted compulsorily. Compared to those admitted voluntarily, the children admitted compulsorily had a higher mean age, were less likely to be living with both parents, and had a higher rate of referral by psychiatric services.

**Table 1:** Demographic and contextual characteristics: means, frequencies, and standard deviations (SD) for all outpatients seen for emergency consultation, and separately for youth not admitted, admitted, and voluntary and compulsory admitted after emergency consultation

	All	Not admitted	Admitted	Voluntary admitted	Compulsory admitted
	*n* = 1194	*n* = 967	*n* = 227	*n* = 137	*n* = 90
Mean age (SD)	14.90 (2.3)	14.75 (2.4)	15.56 (1.6)	15.37 (1.8)	15.84 (1.2)
% Girls	62.8	62.8	63.0	62.8	63.3
**Living situation**					
With two parents	45.0	45.7	41.9	47.4	33.3
With single parent	28.6	27.7	32.2	29.9	35.6
Other	6.5	5.8	9.7	8.8	11.1
Unknown	19.9	20.8	16.3	13.9	20.0
**Location of consultation**					
At home	39.4	42.1	27.8	29.2	25.6
Psychiatric services	14.7	14.6	15.0	18.2	10.0
Police station	8.5	7.0	14.5	12.4	17.8
Other	37.5	36.3	42.7	40.1	46.7
**Referral source**					
General practitioner	37.9	39.7	30.0	36.5	20.0
Psychiatric services	31.2	29.2	40.1	35.8	46.7
Other	30.9	31.1	30.0	27.7	33.3
**Main reason for referral**					
Risk of suicide/self-harm	59.0	58.5	60.8	57.7	65.6
Psychotic symptoms	9.0	7.8	14.5	15.3	13.3
Danger to others	8.1	7.0	12.8	10.9	15.6
Anxiety/depressive symptoms	4.1	4.6	2.2	2.9	1.1
Other	19.8	22.1	9.7	13.1	4.4
**Time of referral**					
Daytime (8 am–6 pm)	65.1	65.7	62.6	62.0	63.3
Evening (6 pm–12 pm)	23.8	24.1	22.5	24.1	20.0
Night (12 pm–8 am)	11.1	10.2	15.0	13.9	16.7

Clinical characteristics are presented in Table 2. The classifications used most were those for externalizing disorders, with a DSM-IV classification indicating relational problems including abuse or an adjustment disorder being registered in over 25% of all consultations.

**Table 2:** Clinical characteristics: frequencies, means, and standard deviations (SD) for all outpatients seen for emergency consultation, and separately for youth not admitted, admitted, and voluntary and compulsory admitted after emergency consultation

	All	Not admitted	Admitted	Voluntary admitted	Compulsory admitted
	*n* = 1194	*n* = 967	*n* = 227	*n* = 137	*n* = 90
**DSM-IV axis I**					
Externalizing disorder	32.4	28.9	47.6	42.3	55.6
Internalizing disorder	26.0	24.9	30.8	35.0	24.4
Relational problems and adjustment disorders	28.3	32.9	8.8	13.9	1.1
Other axis I disorder	13.2	13.3	12.8	8.8	18.9
Comorbidity	32.2	31.2	36.6	39.4	32.2
**Axis II**					
Personality disorder	4.0	3.0	8.4	5.8	12.2
Mental retardation	3.5	3.8	2.2	1.5	3.3
Mean GAF score (SD)	46.7 (11.6)	48.9 (10.7)	37.6 (10.4)	40.3 (9.8)	33.5 (9.9)
In ROPC	62.6	61.8	65.6	65.7	65.6
Prior emergency consultation	17.8	15.5	27.8	20.4	38.9
Known history of inpatient psychiatric treatment	20.6	18.4	30.0	26.3	35.6
Known history of ECA	5.7	4.0	12.8	6.6	22.2
Known history of CO	1.3	1.2	1.8	1.5	2.2
**Moderate or severe ratings on the SPI**					
Suicide risk	34.0	29.0	55.5	46.7	68.9
Danger to others	9.5	6.6	21.6	10.9	37.8
Severity of symptoms	62.8	55.8	92.5	89.1	97.8
Substance abuse or dependence	7.5	6.7	10.6	8.0	14.4
Medical complications	6.4	5.4	11.0	8.8	14.4
Family disruption	45.8	42.4	60.4	56.2	66.7
Vocational impairment	32.2	27.4	52.9	48.2	60.0
Residential instability	15.1	13.2	22.9	20.4	26.7
Lack of motivation for treatment	31.0	26.7	49.3	24.1	87.8
Lack of medical compliance	21.4	18.7	33.0	16.8	57.8
Lack of family involvement	5.6	5.0	8.4	5.8	12.2
Persistence of complaints	53.1	47.5	77.1	71.5	85.6

*GAF* global assessment of functioning, *ROPC* regular outpatient care, *ECA* emergency compulsory admission, *CO* involuntary admission after court order, *SPI* Severity of Psychiatric Illness scale

As shown in the second, third, fourth, and fifth columns of Table 2, the mean severity of problems was greater in children who had been admitted after emergency consultation than in children who had not been admitted, and higher in those admitted compulsorily than in those admitted voluntarily. Other SPI item scores show the same pattern.

### Factors associated with admission (voluntary and compulsory)

Table 3 shows the results of multiple regression analyses for demographic, contextual, and clinical characteristics associated with (voluntary and compulsory) admission after emergency consultations. Correlation analyses to test the bivariate and partial associations between location of consultation, and GAF score, SPI severity of symptoms, and SPI family disruption revealed no significant associations. A greater likelihood of voluntary or compulsory admission was significantly associated with the following: consultation outside the patients’ home, older age, referral by the psychiatric services rather than by a general practitioner, externalizing problems rather than relational problems, poorer global functioning, and severe or moderate SPI scores for severity of symptoms, suicide risk, and family disruption. Receiving regular outpatient care was significantly associated with a lower chance of being admitted.

**Table 3:** Stepwise multiple logistic regression analyses of risk factors associated with outpatient contact versus (voluntary or compulsory) admission (*n* = 1194)

	95% CI for Exp (B)			
	Beta (SE)	Lower	Exp (B)	Upper
Constant	−3.52 (0.94)**			
**Patient and contextual characteristics**				
Age	0.12 (0.05)*	1.01	1.13	1.25
**Location (ref. at home)**				
Police station	1.53 (0.36)**	2.30	4.63	9.32
Psychiatric services	0.67(0.27)*	1.15	1.95	3.33
Other	0.92 (0.25)**	1.54	2.50	4.08
**Referral source (ref. general practitioner)**				
Psychiatric services	0.54 (0.22)*	1.11	1.71	2.62
Other	−0.51 (0.29)	0.34	0.60	1.06
**Clinical characteristics**				
GAF score	−0.71 (0.09)**	0.41	0.49	0.59
**DSM-IV axis I (ref. relational problems and adjustment disorders)**				
Externalizing disorder	0.81 (0.30)*	1.25	2.25	4.04
Internalizing disorder	0.43 (0.31)	0.84	1.53	2.80
Other axis 1 disorder	0.24 (0.36)	0.63	1.27	2.55
In ROPC	−0.46 (0.20)*	0.43	0.63	0.93
**SPI moderate/severe**				
Suicide risk	0.65 (0.19)**	1.32	1.91	2.76
Severity of symptoms	1.46 (0.29)**	2.43	4.29	7.56
Family disruption	0.39 (0.18)*	1.04	1.47	2.10

*R*^2^ = 0.36 (Nagelkerke), AUC = 0.84, 95% CI 0.81–0.87, *p* < 0.0001, Hosmer & Lemeshow Goodness of fit = 7.92, *p* = 0.441

*GAF* global assessment of functioning, *ROPC* regular outpatient care, *SPI* Severity of Psychiatric Illness scale

*P < 0.05, **P < 0.01

### Predictors of compulsory admission as distinct from voluntary admission

Forty-one percent of patients (*n* = 89) in the subgroup of 217 who had been admitted at the age of 13 and older had been admitted compulsorily. Table 4 shows multiple regression analyses results of demographic, contextual, and clinical characteristics associated with voluntary versus compulsory admission after emergency consultation. The following characteristics were significantly associated with a compulsory rather than voluntary admission: prior Emergency Compulsory Admission; severe or moderate SPI scores for suicide risk, danger to others, lack of motivation, and lack of compliance with medication; and all DSM classifications other than those for relational and adjustment disorders. Poorer global functioning increased the likelihood that it was compulsory admission.

**Table 4:** Stepwise multiple logistic regression analyses of risk factors associated with voluntary versus compulsory admission of children age 12 + (*n* = 217)

	95% CI for Exp (B)			
	Beta (SE)	Lower	Exp (B)	Upper
Constant	−6.51 (2.08)**			
**Clinical characteristics**				
DSM-IV axis I (other than relational problems or adjustment disorders)	3.70 (1.83)**	1.12	40.41	1458.79
GAF score	−0.46 (0.23)*	0.40	0.63	1.00
**SPI moderate/severe**				
Suicide risk	1.41 (0.47)**	1.63	4.10	10.30
Danger to others	1.04 (0.53)*	1.00	2.82	7.96
Lack of motivation for treatment	3.13 (0.50)**	8.48	22.77	61.14
Lack of medical compliance	1.46 (0.46)**	1.75	4.31	10.61
Known history of ECA	2.35 (0.74)**	2.44	10.48	45.09

*R*^2^ = 0.69 (Nagelkerke), AUC = 0.94, 95% CI 0.91–0.97, *p* < 0.0001, Hosmer & Lemeshow Goodness of Fit = 5.84, *p* = 0.558

*GAF* global assessment of functioning, *SPI* Severity of Psychiatric Illness scale, *ECA* emergency compulsory admission

*P < 0.05, **P < 0.01

## Discussion

This study was intended to identify possible modifiable factors associated with the voluntary and involuntary admission of children and adolescents who had had a psychiatric emergency consultation.

We found that the chance that a minor would be admitted after an emergency consultation, independently of the legal status, had been smaller if the consultation had taken place in his or her home, and in families that received regular outpatient care before consultation. The chance of admission had been increased if there had been a high level of family disruption.

Not surprisingly, our findings also indicate that the decision to hospitalize had been influenced by clinical factors: children who had been hospitalized after emergency consultation had had more serious symptoms, externalizing problems, and higher scores on the SPI for risk of suicide and severity of problems.

The results of our comparison of factors associated with compulsory and voluntary admission were consistent with what we had expected on the basis of the Dutch law on compulsory admissions: young people had a higher risk of being admitted compulsorily if they had severe psychiatric problems, if they had a higher risk of suicide, if they were a danger to others, and if, due to a lack of motivation and lack of compliance, there were no voluntary alternatives for preventing the danger.

We found two potentially modifiable factors for reducing psychiatric emergency admissions in minors: the location in which the consultation took place and family disruption. As most studies on psychiatric emergency admissions of minors have described the outcome of ED visits in hospitals only, our finding that the risk of psychiatric emergency admission is lower if the consultation takes place in the patient’s home is new and has to be replicated. Based on the association between home assessment and a reduced chance of hospital admission, future studies could investigate whether emergency consultation in the patients’ home indeed is lowering the chance for hospital admission. After emergency consultation at home, it may be easier arranged to have a supervising adult closely monitor the behavior of young person with psychiatric problems.

Our finding that family disruption was associated with the outcome of an emergency consultation is consistent with the literature. If the family system is unable or unwilling to take care of the patient—due to exhaustion, for example—a clinician may opt for hospitalization, independently of the patient’s clinical condition. A reduced chance of admission in families that received regular outpatient care before the emergency consultation suggests that wider use of regular outpatient care may be helpful. Because involvement in evidence-based services is related to both symptom reduction and improvement in functioning [30], it is important to engage youth and families in these services. With timely interventions to decrease family distress, it may be possible to stabilize the situation before it requires more extreme measures. Parents are often best at identifying early behavioral changes in their children, and by having regularly scheduled follow-up appointments that involve monitoring of child’s health and refinement of treatment plans, relapse might be reduced. Also, regular outpatient care may facilitate immediate de-escalation. The development of an individualized, strengths-based safety/crisis plan with the child and family can possibly prevent high levels of family disruption [31]. The fact that an unstable family situation proved to be a predictor of emergency admission adds to the findings of previous studies. Two factors increased the chance that a child would be admitted after emergency consultation: the parents’ absence from the consultation and if a child’s having been adopted [15, 16]. Similarly, the risk of psychiatric hospitalization of children in regular psychiatric outpatient care has been found to be influenced by a family’s level of distress, parental capacity to contain the child, and the primary caregivers’ marital status [32, 33].

Finally, our finding that young people who had been admitted compulsorily had more severe psychiatric problems and a higher suicide risk than those admitted voluntarily is also consistent with prior studies [20, 21]. However, unlike Jendreyschak et al. [21], we did not find that mental retardation was associated with higher compulsory admission rates. This may be because our study included emergency compulsory admissions alone, and not compulsory admissions after a planned court order.

Despite the differences between countries regarding the organization of the emergency services for minors, our findings were generally in line with the literature: a majority of the patients seen for emergency consultation were girls, over half of the patients were already receiving regular outpatient care, and the risk of suicide or self-harm was the commonest reason for referral [2, 15, 34]. In line with the previous studies, patients who were hospitalized after emergency consultation had more severe psychiatric disorders. As described in other studies, we also found older age to be associated with hospitalization after emergency consultation [16, 18, 35].

### Strengths and limitations

A strength of our study is our relatively large sample size, which made it possible to study a comprehensive set of variables. We also used a standardized decision-support tool (the SPI [27, 28]). However, the SPI is not validated for minors. Our study also had four main limitations. First, no information was available on the specific treatment that had been offered before the emergency consultation; unlike adults, children tend to receive mental health services not also from a range of agencies, but also from providers such as school counselors, who, like those at juvenile justice agencies and human service agencies, may not have had mental health training. Second, no information was available on other factors that may have influenced the decision to hospitalize—for example, data about ethnicity were lacking, as was school information, or information on the availability of inpatient beds. Third, DSM-IV classifications were made by clinicians on the basis of a clinical interview and no standard format for assessment was used. Finally, the emergency services in the two regions differed with regard to their organization and staff. However, our variable selection procedure did not find the region of crisis services to have been a predictor of voluntary or compulsory admission.

### Clinical implications

Our findings are clinically relevant, and the association between location of assessment and the chance of hospital admission may suggest that in some cases, psychiatric emergency admissions may be prevented if minors are seen at home rather than in locations such as an ED or psychiatric outpatient unit. Future studies, however, are needed to test this hypothesis. Family interventions in the home situation, including the development of an individualized, strength-based safety/crisis, plan en parent support in families of young people with severe psychopathology and externalizing behavior may prevent family disruption [31], thus making possible for families to cope with a child with severe psychiatric problems in the home situation.

In the past decade, two systematic reviews of intensive home-treatment programs in adult mental healthcare have concluded that intensive treatment models may improve patient satisfaction and reduce hospital use [36, 37]. Earlier research that evaluated different types of intensive home-treatment services for youth provided very little guidance for the development of these services [38]. However, the conclusion of a more recent systematic review of randomized-controlled trials reporting the efficacy of a variety of home-based interventions as alternatives to inpatient care in youth was more promising: inpatient psychiatric hospitalization of youth with severe psychiatric problems did not appear to be more clinically effective than intensive community-based services and these services were associated with lower costs and greater family satisfaction [23]. In our view, intensive outpatient treatment should combine evidence-based treatment of the child’s psychopathology with attention to the parents’ or families’ personal and psychosocial problems, and help to provide parents with the skills and resources which they need for effective parenting children with severe psychopathology. It is not only in emergency situations that outpatient treatment should be intensified: greater attention should also be devoted to parental resources of young people in regular outpatient care. We should be aware that the capacity to cope with a child with psychiatric problems is likely to differ between families, and that taking care of children with severe psychopathology and externalizing behaviour can be exhausting, even in the absence of other family problems.

Finally, since we found the lack of motivation and compliance with medical treatment to be associated with a compulsory rather than voluntary admission, shared decision-making may be required to increase adolescents’ participation in and adherence with psychiatric care [39].

## Conclusions

Seeing minors in their homes and addressing family disruption may help to prevent emergency admissions. Implementation studies should now be used to further test the hypothesis that emergency admissions in young people can be reduced by intensive outpatient treatment that focuses simultaneously on their psychiatric problems, supporting their parents’ needs, and deploying strategies that increase motivation and compliance.

The Medical Ethics Committee confirmed that the Medical Research Involving Human Subjects Act (Dutch acronym: WMO) did not apply to this study, and thus, that no informed consent was required.
